# Risk of Bleeding and Venous Thromboembolism after Colorectal Cancer Surgery in Patients with and without Type 2 Diabetes: A Danish Cohort Study

**DOI:** 10.1055/a-2275-9590

**Published:** 2024-03-26

**Authors:** Frederik Pagh Bredahl Kristensen, Erzsébet Horváth-Puhó, Szimonetta Komjáthiné Szépligeti, Frederikke Schoenfeldt Troelsen, Henrik Toft Sørensen

**Affiliations:** 1 Department of Clinical Epidemiology, Aarhus University Hospital and Aarhus University, Aarhus N, Denmark

**Keywords:** type 2 diabetes, bleeding, venous thromboembolism, colorectal cancer

## Abstract

**Background** Bleeding and venous thromboembolism (VTE) are adverse outcomes after colorectal cancer (CRC) surgery. Type 2 diabetes (T2D) clusters with bleeding and VTE risk factors. We examined the bleeding and VTE risk in patients with T2D undergoing CRC surgery and the prognosis after these adverse outcomes.

**Methods** We conducted a prognostic population-based cohort study of 48,295 patients with and without T2D undergoing surgery for incident CRC during 2005 to 2019. Patients with T2D were diagnosed in a hospital setting or had redeemed a glucose-lowering drug prescription; the remaining cohort was patients without diabetes. We estimated the 30-day and 1-year risks of bleeding and VTE and used a Fine–Gray model to compute age-, sex-, and calendar year-adjusted subdistribution hazard ratios (SHRs). The Kaplan–Meier method was used to calculate 1-year mortality after bleeding or VTE.

**Results** Within 30 days after CRC surgery, the risk of bleeding was 2.7% in patients with T2D and 2.0% in patients without diabetes (SHR: 1.30 [95% confidence interval [CI]: 1.10–1.53]). For VTE, the 30-day risks were 0.6% for patients with T2D and 0.6% for patients without diabetes (SHR: 1.01 [95% CI: 0.71–1.42]). The SHRs for bleeding and VTE within 1 year after CRC surgery were similar. The 1-year mortality was 26.0% versus 24.9% in the bleeding cohort and 25.8% versus 27.5% in the VTE cohort for patients with T2D versus without diabetes, respectively.

**Conclusion** Although absolute risks were low, patients with T2D have an increased risk of bleeding but not VTE after CRC surgery.

## Introduction

Colorectal cancer (CRC) is the third most common cancer worldwide and a leading cause of morbidity and mortality. 1 2 Patients with type 2 diabetes (T2D) have a 30% higher risk of CRC than those without diabetes. 3 It is expected that the incidence of T2D will continue to increase globally and a larger proportion of CRC patients will have T2D. 4 5 Although survival after CRC surgery has improved over the past decade, 6 7 T2D is associated with a more than 50% elevated mortality after CRC surgery, 5 8 9 10 11 12 possibly because of an elevated risk of T2D-associated adverse outcomes, such as bleeding and thromboembolism. 5 8 9 12 13

Venous thromboembolism (VTE) is a potential fatal peri- and postoperative outcome, with a 30-day risk of 0.3 to 3.0% despite treatment with thromboprophylaxis. 14 15 16 17 18 Thromboprophylaxis has benefits and drawbacks, in that the risk of VTE must be balanced against the risk of bleeding. 19 However, striking this balance in patients with T2D can be challenging. T2D is associated with obesity, hypercoagulability, and microvascular damage, all of which may increase the risk of VTE. 20 21 Simultaneously, T2D is associated with an elevated prevalence of hypertension, liver cirrhosis, and chronic kidney disease and these factors may increase the bleeding risk. 20 21 22 23 Accordingly, T2D has been linked to a relative risk elevation of 20% for bleeding 22 24 25 26 27 and 40% for VTE. 20 21 However, evidence concerning the risk of bleeding and VTE in patients with coexisting CRC and T2D, and the prognostic impact of these adverse outcomes, is lacking. Such knowledge is essential to guide clinicians in optimizing thromboprophylaxis duration and decreasing the bleeding risk among the most vulnerable patients with CRC, including those with T2D. 15 28 29 30

Therefore, we conducted a prognostic population-based cohort study with two aims. First, we assessed the absolute and relative risks of hospital-diagnosed bleeding and VTE after CRC surgery in patients with and without T2D. Second, among patients who underwent surgery for CRC, we examined the 1-year mortality after bleeding or VTE in those with T2D compared to those without diabetes.

## Methods

### Study Design, Setting, and Registries

We used Danish health and administrative registries to conduct a population-based cohort study. The tax-funded Danish National Health Service provides access to free medical care for all legal residents of Denmark. 31 The unique 10-digit civil registration number issued at birth or migration to all Danish residents enabled individual-level linkage across registries. 31 We used data from four registries: the Danish Civil Registration System, containing dates of birth, death, and emigration since 1968; the Danish National Patient Registry covering all Danish hospitals, containing inpatient hospitalizations since 1977, and outpatient hospital clinic contacts and surgical procedures (Nordic Medico-Statistical Committee [NOMESCO]) since 1996 32 ; the Danish Cancer Registry, containing dates of CRC diagnosis and tumor characteristics since 1987 33 ; and the Danish National Prescription Registry, containing reimbursed drug prescriptions redeemed at community pharmacies since 1995. 34 All diagnosis and procedure codes used in the current study are listed in Supplementary Tables S1 to S4 .

This study was approved by the Danish Health Data Authority and registered at Aarhus University on behalf of the Danish Data Protection Agency (No 2016-051-000001/812). According to Danish legislation, separate ethical committee approval is not needed for register-based studies.

### Study Cohort

Supplementary Fig. S1 presents a flowchart of the study cohort. We identified 67,530 individuals in the Danish Cancer Registry with incident CRC recorded between January 1, 2005 and December 31, 2019. We included all patients with a first-time NOMESCO code for CRC surgery recorded within 90 days after a CRC diagnosis ( *N* = 48,880 [72% of all patients with a CRC diagnosis code]). We excluded patients not registered in the Danish Civil Registration System within 1 year before CRC surgery ( *N* = 270) and those with malignant neoplasms of the appendix ( *N* = 10).

### Definition of Type 2 Diabetes

We used the Danish National Patient Registry and the Danish National Prescription Registry to identify patients with T2D. Patients with T2D were diagnosed in a hospital setting or/and redeemed at least one prescription for a glucose-lowering drug any time before the CRC surgery date. The identification of patients with diabetes has been validated in a Danish setting, with a positive predictive value of 89% for this algorithm. 35 Patients treated with insulin monotherapy any time before CRC surgery were excluded to limit the number of patients with other diabetes subtypes, such as type 1 diabetes or latent autoimmune diabetes in adults ( *N* = 305). 36 The remaining cohort was defined as patients without diabetes.

### Study Outcomes

For the first study aim, the primary endpoints were hospital-diagnosed bleeding and VTE, according to data retrieved from the Danish National Patient Registry. We defined bleeding as a composite of primary or secondary discharge diagnosis codes for intracranial, respiratory, gastrointestinal, and urinary tract bleeding, bleeding-related anemia, or bleeding necessitating reoperation. 37 38 Similarly, we defined VTE as a composite of primary or secondary discharge diagnosis codes for deep vein thrombosis and pulmonary embolism. As we included both prevalent and incident events, patients with a prior VTE or bleeding event were not excluded from the study cohort. Diagnoses were coded during inpatient hospitalization, emergency department contact, or outpatient clinic contact, with one exception: VTE diagnosis codes from emergency department contacts alone were omitted because the diagnosis code is used as a tentative working diagnosis in that setting. 39 For the second study aim, we retrieved information on all-cause mortality from the Civil Registration System.

### Covariates

Information on age and sex at CRC surgery was ascertained from the Danish Civil Registration System. The calendar period of surgery and stage was ascertained from the Danish Cancer Registry. The following treatments, surgical procedures, and comorbidities were ascertained from the Danish National Patient Registry to characterize CRC patients with and without T2D: neoadjuvant chemotherapy, type of first surgical approach (open/laparoscopic/endoscopic), resection site (total colectomy, partial colectomy, rectal resection, and endoscopic surgeries), comorbidities included in the Charlson comorbidity index (CCI) score, obesity, hypertension, atrial fibrillation, risk factors for bleeding (chronic liver disease and chronic kidney failure), and prior bleeding and/or VTE events. We further obtained information on cardiovascular disease (myocardial infarction, angina pectoris, percutaneous coronary revascularization, congestive heart failure, cerebrovascular disease, thrombolysis/thrombectomy for stroke, peripheral artery disease, and lower limb revascularization/amputation) and lifestyle factors, by using proxy measures of alcohol use (diseases associated with alcohol use disorder or treatment with disulfiram) and smoking (chronic obstructive pulmonary disease or use of bronchodilators). We calculated CCI scores (excluding diagnoses of diabetes and CRC) and categorized patients into three subgroups according to their estimated score: 0 (low), 1 to 2 (moderate), or +3 (high). 40 Information on medication associated with bleeding and VTE risk was obtained from the Danish National Prescription Registry, including thromboprophylaxis (vitamin K antagonists, direct oral anticoagulants, and heparins), platelet aggregation inhibitors (aspirin and adenosine-diphosphate receptor antagonists), nonsteroidal anti-inflammatory drugs, oral corticosteroids, antidepressants, and proton pump inhibitors. We also obtained information on glucose-, blood pressure-, and lipid-lowering therapies. The look-back period was not restricted for hospital discharge diagnosis codes, surgical codes, and procedure codes but was limited to 1 year for all drug use.

### Statistical Analyses

We characterized patients according to the abovementioned covariates, stratified by the presence of T2D. We followed patients with T2D and those without diabetes from the date of CRC surgery until the first occurrence of a primary endpoint, emigration, death, or the study end on April 1, 2021, thus ensuring at least 1 year of follow-up. For absolute risks, we used the cumulative incidence function, considering death as a competing event. We calculated crude risk differences and used Fine–Gray subdistribution hazard regression models to estimate age-, sex-, and calendar-time-adjusted subdistribution hazard ratios (SHRs) for bleeding and VTE, using patients without diabetes as the reference cohort. We also calculated cause-specific hazard ratios (HRs) using Cox proportional hazards regression to provide complementary information in a competing risk setting, as previously recommended. 41 We considered time elapsed since the date of CRC surgery as the underlying time scale. The main analysis provided 0–30-day and 0–1-year estimates based on guidelines recommending thromboprophylaxis for up to 28 days after CRC surgery. 19 28 The analyses were also performed for each individual outcome included in the bleeding and VTE composite outcome to identify the underlying main driver.

We assessed heterogeneity in bleeding and VTE risk by calculating 1-year risks, risk differences, and SHRs in predefined subgroups to identify the primary factors influencing bleeding and VTE risk. This analysis included stratification on factors that may act as intermediate factors between T2D and risk of VTE or bleeding. We included age (50–59, 60–69, 70–79, or 80+ years); sex; calendar year of CRC surgery (2005–2009, 2010–2014, or 2015–2019); tumor stage (localized, regional, metastatic, or unknown); type of first surgical approach; obesity; hypertension; CCI score (0, 1–2, or 3 + ); atrial fibrillation; chronic liver disease; chronic kidney disease; cardiovascular disease; prior bleeding events; prior VTE events; and use of statins, platelet aggregation inhibitors, and thromboprophylaxis.

To examine bleeding- and VTE-related mortality, we followed patients who encountered a bleeding or VTE event within 1 year after CRC surgery until death, emigration, or the study end on April 1, 2021. We used the Kaplan–Meier estimator to calculate 1-year mortality and a Cox proportional hazards regression analysis to compute age-, sex-, and calendar-time-adjusted mortality rate ratios.

### Additional Analyses

We performed two additional analyses. First, to eliminate the short-term impact of postoperative thromboprophylaxis, we estimated risk of bleeding and VTE for the period from 31 days until 365 days. Second, to ensure the sensitivity of our outcome definitions, we added prespecified primary and secondary discharge diagnosis and procedure codes to our composite endpoints. These codes included postoperative bleeding, treatment with blood products, postoperative thromboembolism, and unspecified thrombosis, all obtained from the Danish National Patient Registry.

## Results

### Description of the Study Cohort

Table 1 and Supplementary Table S5 present the patient characteristics on the date of CRC surgery for both patients with T2D and those without diabetes. After application of the exclusion criteria, 48,295 patients underwent CRC surgery, among whom 6,305 (13%) had T2D. Patients with T2D versus those without diabetes showed an almost similar age distribution (73 vs. 71 years), type of first surgical approach (e.g., laparoscopic surgery rate of 38% in both cohorts), time from diagnosis of CRC until surgery (median 6 days in both cohorts), tumor stage (e.g., metastatic cancer in 15% vs. 16%), and prior VTE prevalence (5% vs. 4%). In contrast, patients with T2D were more likely to be men (62% vs. 52%); had a higher burden of comorbidities (e.g., CCI score of +3: 19% vs. 11%); and had a higher prevalence of prior bleeding events (23% vs. 17%), hypertension (73% vs. 39%), and obesity (18% vs. 4%). Patients with T2D also had higher usage rates of thromboprophylaxis (15% vs. 8%), platelet aggregation inhibitors (47% vs. 23%), and statins (67% vs. 25%).

**Table 1:** Characteristics of patients with type 2 diabetes or without diabetes at the time of colorectal cancer surgery

	Type 2 diabetes	No diabetes
*N*	6,305 (100.0%)	41,990 (100.0%)
Male	3,910 (62.0%)	21,735 (52.0%)
Age, median (Q1–Q3)	73.4 (67.3–79.4)	71.4 (63.7–78.7)
Calendar year		
2005–2009	1,545 (24.0%)	12,695 (30.0%)
2010–2013	2,085 (33.0%)	13,705 (33.0%)
2014–2019	2,675 (42.0%)	15,585 (37.0%)
Days from CRC diagnosis until surgery, median (Q1–Q3)	6.0 (1.0–20.0)	6.0 (1.0–19.0)
Tumor stage		
Localized	2,765 (44.0%)	18,020 (43.0%)
Regional	1,585 (25.0%)	10,905 (26.0%)
Metastatic	915 (15.0%)	6,535 (16.0%)
Unknown	1,040 (17.0%)	6,530 (16.0%)
Type of first surgical approach		
Open	2,380 (38.0%)	15,730 (37.0%)
Laparoscopic	2,375 (38.0%)	15,875 (38.0%)
Endoscopic	1,555 (25.0%)	10,385 (25.0%)
Tumor resection site		
Total colectomy	155 (2.0%)	1,020 (2.0%)
Partial colectomy	3,475 (55.0%)	21,885 (52.0%)
Rectal resection	1,120 (18.0%)	8,665 (21.0%)
Other colorectal surgeries	1,555 (25.0%)	10,420 (25.0%)
Neoadjuvant chemotherapy	510 (8.0%)	3,215 (8.0%)
Hypertension	4,625 (73.0%)	16,165 (39.0%)
Obesity	1,140 (18.0%)	1,765 (4.0%)
Alcohol use disorders	450 (7.0%)	1,920 (5.0%)
Smoking	800 (13.0%)	3,595 (9.0%)
CCI score		
0	2,620 (42.0%)	24,375 (58.0%)
1–2	2,495 (40.0%)	13,055 (31.0%)
3+	1,190 (19.0%)	4,560 (11.0%)
Prior bleeding episodes	1,460 (23.0%)	7,225 (17.0%)
Prior VTEs	325 (5.0%)	1,770 (4.0%)
Atrial fibrillation	1,130 (18.0%)	4,160 (10.0%)
Chronic kidney failure	650 (10.0%)	1,180 (3.0%)
Medications		
Platelet aggregation inhibitors	2,990 (47.0%)	9,530 (23.0%)
Aspirin	2,730 (43.0%)	8,400 (20.0%)
Thromboprophylaxis	930 (15.0%)	3,400 (8.0%)
Non-aspirin NSAIDs	1,405 (22.0%)	8,765 (21.0%)
Antidepressants	895 (14.0%)	4,445 (11.0%)
Statins	4,200 (67.0%)	10,295 (25.0%)
Loop diuretics	1,550 (25.0%)	4,030 (10.0%)
ACE inhibitors/ARBs	4,165 (66.0%)	12,495 (30.0%)
Thiazides	1,330 (21.0%)	6,385 (15.0%)
Proton pump inhibitors	2,130 (34.0%)	10,465 (25.0%)

Abbreviations: ACE inhibitors/ARBs, angiotensin-converting enzyme inhibitors/angiotensin receptor blockers; CCI, Charlson comorbidity index; CRC, colorectal cancer; NSAIDs, nonsteroidal anti-inflammatory drugs; VTE, venous thromboembolism.

Note: Definitions of covariates are provided in Supplementary Tables S1–S4 . Supplementary Table S5 provides an extended table of patient characteristics. Numbers have been rounded to the nearest 5, in accordance with Danish health data legislation.

### Type 2 Diabetes and Risk of Bleeding after Surgery for Colorectal Cancer

**Fig. 1:**
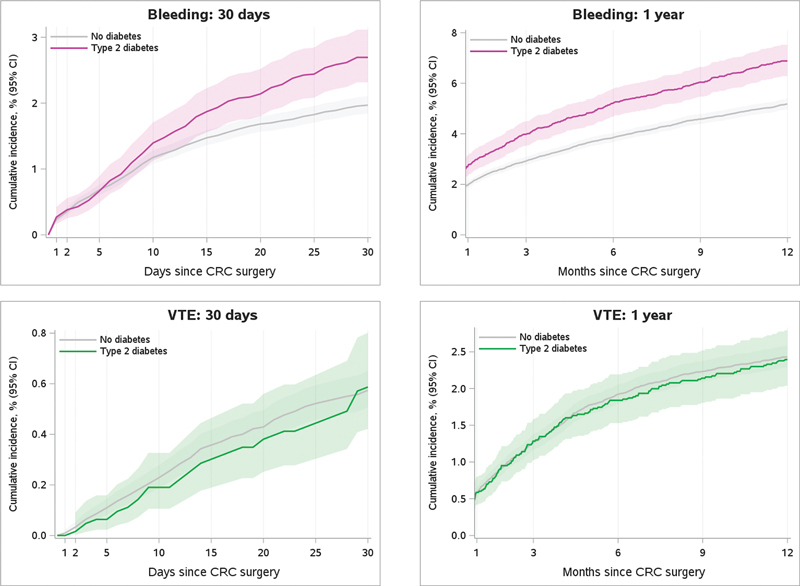
**30-day and 1-year risks of bleeding and venous thromboembolism after colorectal cancer surgery according to type 2 diabetes presence.** Patients were followed from the date of surgery until the first occurrence of an event of interest, emigration, death, or study end (April 1, 2021), whichever came first. The Aalen–Johansen estimator was used to estimate 30-day and 1-year risks of bleeding and VTE, by considering death as a competing event. CI, confidence interval; CRC, colorectal cancer; VTE, venous thromboembolism.

Within 30 days after CRC surgery, we identified 995 cases of hospital-diagnosed bleeding ( Table 2 ). The absolute risks were 2.7% (95% confidence intervals [CIs]: 2.3–3.1) for patients with T2D and 2.0% (95% CI: 1.8–2.1) for patients without diabetes, corresponding to an age-, sex-, and calendar-year-adjusted SHR of 1.30 (95% CI: 1.10–1.53). Within the first year after CRC surgery, the absolute risks were 6.9% (95% CI: 6.3–7.5) for patients with T2D and 5.2% (95% CI: 5.0–5.4) for patients without diabetes, thus yielding an increased adjusted SHR of 1.26 (95% CI: 1.14–1.40) ( Fig. 1 and Table 2 ). The risk of bleeding was driven primarily by bleeding from the lower gastrointestinal tract ( Supplementary Table S6 ). In the subgroup analyses, the 1-year absolute risk of bleeding increased with age and was highest among patients with CCI scores of +3, chronic liver disease, chronic kidney disease, atrial fibrillation, prior bleeding episodes, and thromboprophylaxis ( Supplementary Table S7 ). Compared with patients without diabetes, the adjusted SHRs were attenuated among patients with T2D with a disease or treatment associated with increased bleeding risk, including chronic kidney disease, obesity, metastatic cancer, use of platelet aggregation inhibitors, and prior bleeding episodes ( Supplementary Table S7 ).

**Table 2:** Risks, risk differences, and adjusted associations of bleeding and venous thromboembolism for patients with type 2 diabetes and without diabetes after colorectal cancer surgery

		N/events	Risk (95% CI)	Risk difference (95% CI)	Age-, sex-, and calendar year-adjusted SHR (95% CI)	Age-, sex-, and calendar year-adjusted cause-specific HR (95% CI)
Bleeding events						
30 days	No diabetes	41,990/825	2.0 (1.8–2.1)		Reference	Reference
	Type 2 diabetes	6,305/170	2.7 (2.3–3.1)	0.7 (−0.1–1.6)	1.30 (1.10–1.53)	1.30 (1.10–1.54)
1 year	No diabetes	41,990/2,175	5.2 (5.0–5.4)		Reference	Reference
	Type 2 diabetes	6,305/435	6.9 (6.3–7.5)	1.7 (0.4–3.0)	1.26 (1.14–1.40)	1.28 (1.16–1.43)
Venous thromboembolism						
30 days	No diabetes	41,990/240	0.6 (0.5–0.6)		Reference	Reference
	Type 2 diabetes	6,305/35	0.6 (0.4–0.8)	0.0 (−0.3–0.5)	1.01 (0.71–1.42)	1.01 (0.72–1.44)
1 year	No diabetes	41,990/1020	2.4 (2.3–2.6)		Reference	Reference
	Type 2 diabetes	6,305/150	2.4 (2.0–2.8)	−0.0 (−0.8–0.8)	0.96 (0.81–1.14)	0.98 (0.82–1.16)

Abbreviations: CI, confidence interval; HR, hazard ratio; PY, person-year; SHR, sub-distribution hazard ratio.

Note: Patients were followed from the date of surgery until the first occurrence of an event of interest, emigration, death, or study end (April 1, 2021), whichever came first. The Aalen–Johansen estimator was used to estimate 30-day and 1-year risks of bleeding and venous thromboembolism, by considering competing risk of death. Numbers have been rounded to the nearest 5, in accordance with Danish health data legislation.

### Type 2 Diabetes and Risk of Venous Thromboembolism after Surgery for Colorectal Cancer

Within 30 days after CRC surgery, the VTE risk was low overall and was similar between patients with T2D (0.6% [95% CI: 0.4–0.8]) versus without diabetes (0.6% [95% CI: 0.5–0.6]) ( Fig. 1 and Table 2 ). Within the first year after CRC surgery, the absolute risk of VTE increased to 2.4% (95% CI: 2.0–2.8) for patients with T2D and to 2.4% (95% CI: 2.3–2.6) for patients without diabetes (age-, sex-, and calendar-year-adjusted SHR of 0.96 [95% CI: 0.81–1.14]). For the 30-day and 1-year risk estimates, VTE occurrence was driven primarily by the number of pulmonary embolism events ( Supplementary Table S6 ). In the subgroup analyses, the 1-year absolute VTE risk was particularly elevated in patients with prior VTE (T2D: 12.0% [95% CI: 8.8–15.8]; no diabetes: 14.9% [95% CI: 13.3–16.6]), whereas the risks in other subgroups were comparable to those in our main analysis. Adjusted SHRs for VTE were comparable across patient subgroups ( Supplementary Table S8 ).

### Mortality after Bleeding and Venous Thromboembolism

**Fig. 2:**
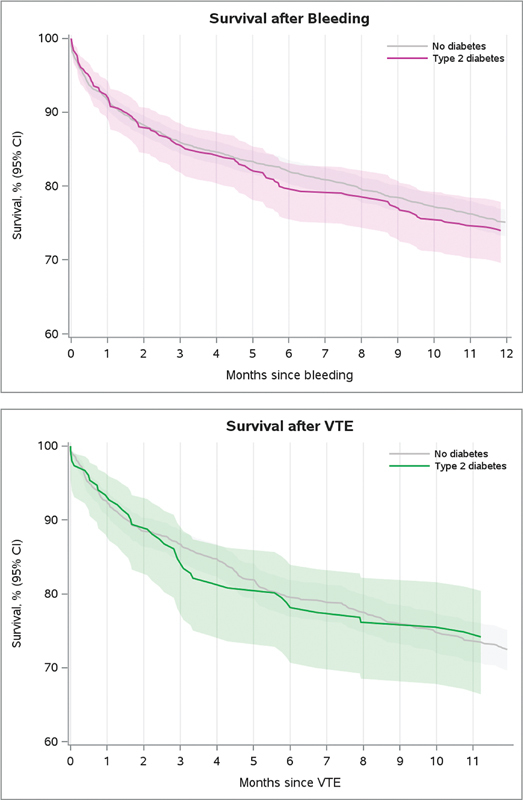
**1-year survival for patients with a bleeding or venous thromboembolism event within 1 year after colorectal cancer surgery.** Patients were followed from a bleeding/VTE outcome until 1 year of follow-up, death, or study end (April 1, 2021), whichever came first. The Kaplan–Meier estimator was used to estimate 1-year mortality. CI, confidence interval; VTE, venous thromboembolism.

Fig. 2 and Supplementary Table S9 present mortality after a bleeding or VTE event. In patients with a bleeding event within 1 year after CRC surgery, the 1-year mortality was 26.0% (95% CI: 22.2–30.4) for patients with T2D and 24.9% (95% CI: 23.2–26.8) for patients without diabetes (adjusted cause-specific HR of 1.46 [95% CI: 0.59–3.60]). The 1-year mortality after a VTE event was 25.8% (95% CI: 19.6–33.6) in patients with T2D and 27.5% (95% CI: 24.9–30.4) in patients without diabetes (adjusted cause-specific HR of 1.02 [95% CI: 0.73–1.43]).

### Additional Analyses

The risks of bleeding and VTE during 31 to 365 days of follow-up were comparable to the 1-year risk estimate ( Supplementary Table S10 ). Use of a more extensive definition of bleeding, including diagnosis codes for postoperative bleeding and treatment with blood products, increased the 30-day absolute risk from 2.7% (95% CI: 2.3–3.1) to 5.9% (95% CI: 5.4–6.5) for patients with T2D and from 2.0% (95% CI: 1.8–2.1) to 4.6% (95% CI: 4.4–4.8) for patients without diabetes ( Supplementary Table S11 ). A similar doubling in absolute risks occurred for 1-year absolute risks ( Supplementary Table S11 ). However, the adjusted SHRs remained unchanged (30-day SHR: 1.30 [95% CI: 1.16–1.45]; 1-year SHR: 1.31 [95% CI: 1.21–1.41]). A more extensive definition of VTE changed neither the absolute estimates nor the SHRs ( Supplementary Table S11 ).

## Discussion

In this population-based cohort study, we investigated 30-day and 1-year risks of hospital-diagnosed bleeding and VTE among 48,295 patients with and without T2D who underwent CRC surgery. Although the absolute risk estimates were low, presence of T2D was associated with a 28% higher 1-year risk of bleeding than for patients without diabetes. However, the association was attenuated in subgroups with high bleeding risk, suggesting that factors other than T2D may contribute to the increased bleeding risk. Likewise, we observed low 30-day and 1-year risks of VTE after CRC surgery and no association between T2D and VTE. Among patients who experienced a bleeding or VTE event, approximately one in four patients (25% risk) died within 1 year.

Our findings align with previous evidence. Prior studies have suggested that the presence of diabetes is associated with a 20% increase in bleeding risk in patients with any cancer 27 or patients treated with thromboprophylaxis or aspirin. 22 23 24 26 However, these studies did not specifically examine CRC patients, they included all diabetes subtypes rather than focusing on T2D, and did not examine the impact of potential underlying bleeding factors. Among patients who underwent CRC surgery, we found a weak association between T2D and bleeding. This increased bleeding tendency might be explained by an accumulation of risk factors for bleeding in patients with T2D compared to those without diabetes, suggesting that T2D per se is not associated with bleeding risk. 22 This hypothesis was further confirmed by our subgroup analyses, in which the association for T2D was attenuated among patients with chronic kidney disease, obesity, metastatic cancer, and use of platelet aggregation inhibitors. Thus, the increased prevalence of these risk factors among patients with T2D might explain the overall association observed between T2D and bleeding. This is further supported by a United Kingdom cohort study suggesting that the burden of adverse postoperative outcomes in patients with CRC and T2D was highest when accompanied by one or more T2D complications. 12 In contrast, T2D patients without complications had a prognosis similar to that of patients without diabetes. Hence, the risk of complications, such as bleeding or VTE, may depend on the burden of T2D complications and comorbidities, rather than the T2D condition itself.

The risk of VTE in our study was consistent with the 30-day VTE rate reported in randomized controlled trials. 30 Prior studies have indicated that T2D increases the risk of VTE. 20 21 Mechanistically, obesity and other metabolic syndrome components are associated with hypercoagulability and microvascular damage. 20 21 Other risk factors for VTE may include cardiovascular diseases and cancer, which often cluster among patients with T2D. 3 20 42 The null association observed between T2D and VTE in our study might be explained by a high user rate of thromboprophylaxis in the 30 days after CRC surgery. Another explanation might be that our study cohort encompassed patients with an equal distribution of the strongest VTE risk factors such as presence of cancer, surgery, and long bed rest thereby weakening the association between T2D and VTE. 19

We showed that bleeding and VTE outcomes were associated with a comparable 1-year mortality between patients with T2D and those without diabetes. While it is well known that coexisting VTE and cancer is associated with an up to fourfold increased mortality compared with cancer patients without VTE, 43 mortality after bleeding as well as the impact of T2D is unknown. A recent Danish cohort study reported that colon cancer patients who experienced a VTE after the cancer diagnosis had a threefold higher 1-year mortality than cancer patients without VTE (mortality 42.6% for VTE cohort vs. 13.8% for comparison cohort). 43 The lower 1-year mortality observed in our study could be explained by our cohort being restricted to patients with an early VTE event (maximum 1 year after CRC surgery). Although it is crucial to prevent VTE, 15 18 19 28 30 our results suggest that preventing bleeding complications may be equally important in enhancing survival after CRC surgery. Yet, the lack of clinical details and the inability to validly ascertain causes of death in the present study precludes conclusions about the potential causal relation between T2D and mortality from bleeding or VTE.

Our results are of clinical interest for several reasons. First, due to the rising number of individuals with T2D, our study improves the understanding of patient characteristics and the course of disease for patients with CRC and preexisting T2D. 4 5 36 Second, improving primary prevention of VTE in CRC survivors requires identification of high-risk individuals, for whom the potential benefits of thromboprophylaxis outweigh the potential risks of bleeding, and striking this balance in T2D may be difficult. Our study suggested that the risks of bleeding and VTE depend on the presence of other risk factors than T2D. For example, risk of bleeding increased with age and with the presence of comorbidities including chronic liver disease, chronic kidney disease, and prior bleeding episodes, whereas the VTE risk was highest for patients with prior VTE. These factors could be considered to capture individual risk profiles for both VTE and bleeding for optimizing thromboprophylaxis duration. 15 18 19 28 30

### Strength and Limitations

The strengths of our study include its large sample size, its design implemented in a population-based setting with universal health care, and the use of high-quality nationwide health and administrative registries with complete follow-up. 44

Our study also has limitations. First, using hospital-based diagnoses might have led to underestimation of the absolute risk of bleeding and VTE. The diagnosis codes for lower gastrointestinal bleeding, anemia caused by bleeding, and VTE in the Danish National Patient Registry, respectively, have been validated with a positive predictive value >90% but unknown sensitivity. 37 38 45 Although the validity of relative risk estimates depends on predictive values, the validity of absolute risk estimates depends on the sensitivity of the outcome. Less severe bleeding, including blood loss during surgery, is not recorded in the Danish National Patient Registry. 32 46 To increase sensitivity, we included treatment with blood products as a secondary outcome, which showed a twofold greater absolute bleeding risk than that in the main analysis, but similar adjusted SHRs. Thus, lower sensitivity might not have influenced the SHRs. 47 Third, our characterization of CRC patients lacked detailed information about socio-behavioral factors, including smoking, alcohol intake, and obesity. Furthermore, we lacked clinical information on the extent of bleeding and thrombosis; severity of underlying diseases; presence of clinical markers for pro-bleeding and pro-thrombotic disorders, including international normalized ratio or thrombocyte level; and medications administered during hospital stays. 22 The lack of information on medications administered during hospitalization prevented a full assessment of thromboprophylaxis use after CRC surgery.

In conclusion, although the absolute risk was low, patients with CRC and T2D may have a slightly elevated relative risk of bleeding, but not VTE, after CRC surgery. We found no difference in risk of mortality after bleeding and VTE in patients with and without T2D.
