# Supplementary material for: Risk of Bleeding and Venous Thromboembolism after Colorectal Cancer Surgery in Patients with and without Type 2 Diabetes: A Danish Cohort Study
Source: TH Open. 2024 Mar 26;8(1):e146–54. doi: 10.1055/a-2275-9590 (PMC10965306; doi:10.1055/a-2275-9590)
Supplement: Supplementary file 1 — Supplementary Material [file 10-1055-a-2275-9590-s23120052.pdf]

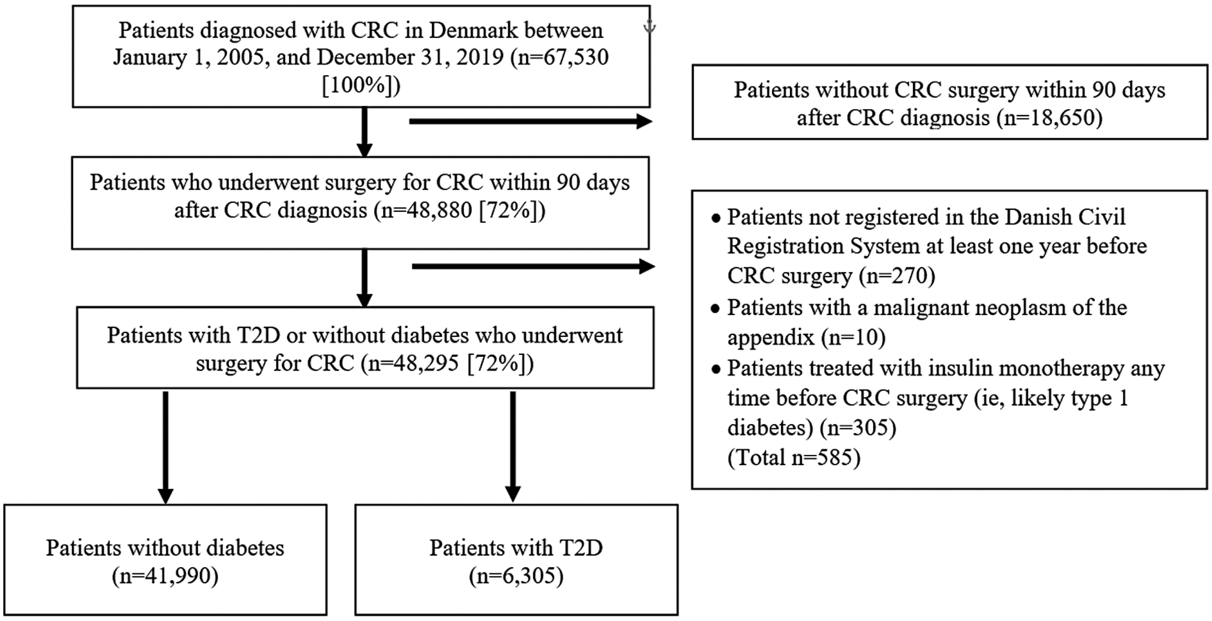

Supplementary Fig. S1 Flowchart of the study population.

 © 2024. The Author(s).

Supplementary Table S1 Codes used to define the study cohort, exposure, and outcomes

Variable	ICD-10/procedure/surgical codes	ATC codes	Definition	Registry
Study cohort	C18–C20 (except C18.1)		First record of CRC	Danish Cancer Registry
Surgery for CRC	KJFH KJFB KJGB KJFA KJGA		Surgery performed within 90 days after CRC diagnosed	Danish National Patient Registry
Type 2 diabetes	ICD-10: E10–E14, O24 [except O244], G632, H360, N083	A10	• Primary or secondary diagnosis of diabetes recorded in a hospital inpatient, emergency department, or outpatient setting any time before CRC surgery<br>• Redeemed prescription for a glucose-lowering drug any time before CRC surgery	Danish National Patient Registry and Danish National Prescription Registry
Exclusion criteria				
Type 1 diabetes		A10A	Insulin use (ATC code: A10A) and no use of non-insulin GLDs (ATC code: A10B or A10X) any time before CRC surgery	Danish National Prescription Registry
Outcomes				
Hospital-diagnosed bleeding	Composite endpoint of bleeding from the respiratory tract, upper/lower gastrointestinal tract, and urinary tract; anemia from bleeding intracerebral bleeding; and reoperation due to bleeding (see codes below)		Primary or secondary discharge diagnosis codes recorded during an inpatient hospitalization, emergency department contact, or outpatient clinic contact	Danish National Patient Registry
Respiratory tract bleeding	R04.0, R04.2			Danish National Patient Registry
Upper gastrointestinal tract bleeding	I85.0, K25.0, K25.2, K25.4, K25.6, K26.0, K26.2, K26.4, K26.6, K27.0, K27.2, K27.4, K27.6, K28.0, K28.2, K28.4, K28.6, K29.0			Danish National Patient Registry
Lower gastrointestinal tract bleeding	K62.5, K92.0–K92.2<br>K638C			Danish National Patient Registry
Urinary tract bleeding	R31.9, N02			Danish National Patient Registry
Anemia from bleeding	D62			Danish National Patient Registry
Intracerebral bleeding	I60, I61, I62, S06.4–S06.6			Danish National Patient Registry
Reoperation due to bleeding	KJWD, KJWE			Danish National Patient Registry
Postoperative bleeding or hematoma *(used in sensitivity analysis)*	T810		Primary or secondary discharge diagnosis codes recorded during an inpatient	Danish National Patient Registry

(*Continues*)

 © 2024. The Author(s).

Supplementary Table S1 (*Continued*)

Variable	ICD-10/procedure/surgical codes	ATC codes	Definition	Registry
			hospitalization, emergency department contact, or outpatient clinic contact	
Treatment with blood products *(used in sensitivity analysis)*	BOQ		Treatment codes recorded during an inpatient hospitalization, emergency department contact, or outpatient clinic contact	Danish National Patient Registry
Venous thromboembolism	Composite endpoint of deep vein thrombosis and pulmonary embolism (see codes below)		Primary discharge diagnosis codes recorded during an inpatient hospitalization, or outpatient clinic contact	Danish National Patient Registry
Deep vein thrombosis	I80.1–I80.3			Danish National Patient Registry
Pulmonary embolism	I26			Danish National Patient Registry
Postoperative thromboembolism *(used in sensitivity analyses)*	T817 (except T817Y and T817X)		Primary or secondary discharge diagnosis codes recorded during an inpatient hospitalization, emergency department, or outpatient clinic contact	Danish National Patient Registry
Unspecified thrombosis *(used in sensitivity analyses)*	I828, I829		Primary or secondary discharge diagnosis codes recorded during an inpatient hospitalization, emergency department, or outpatient clinic contact	Danish National Patient Registry

Abbreviations: ATC, Anatomical Therapeutic Chemical; CRC, colorectal cancer; ICD-10, International Classification of Diseases, Tenth Revision.

 © 2024. The Author(s).

Supplementary Table S2 Definitions of covariates

	ICD 10	ICD 8	ATC codes	Definition	Registry
**Resection type**				Resection type performed on the day of CRC surgery (see codes above)	Danish National Patient Registry
Total colectomy	KJFH				
Partial colectomy	KJFB				
Rectal resection	KJGB				
Endoscopic surgeries	KJFA, KJGA				
**Tumor stage**				Tumor classification at CRC diagnosis	Danish Cancer Registry
Localized	T1–4, x; N0; M0<br>T1–2; N0; Mx<br>T1–1; Nx, M0, x				
Regional	T1–4, x; N1–3, M0				
Metastatic	T1–4, x; N1–3, M1, x<br>T1–4, x; N0; M1<br>T1–4, x; Nx; M1				
Unknown	T2–4, x; Nx, M0, x<br>T3–4, x; N0; Mx				
**Type of first surgical approach**				Type of surgery performed the day of CRC surgery (see codes above)	Danish National Patient Registry
Laparoscopic	Surgical codes: KJFA17, KJFA87, KJFA88, KJFA61, KJFA71, KJFA74, KJFA81, KJFA84, KJFA97 KJFH01, KJFH11, KJFH21, KJFH24, KJFH25, KJFH31, KJFH34, KJFB01, KJFB21, KJFB31, KJFB34, KJFB41, KJFB44, KJFB47, KJFB51, KJFB54, KJFB57, KJFB61, KJFB64, KJFB97, KJGA97, KJGB01, KJGB04, KJGB11, KJGB31, KJGB33, KJGB34, KJGB37, KJGB61, KJGB97				
Endoscopic	Surgical codes: KJFA00, KJFA02, KJFA05, KJFA10, KJFA12, KJFA15, KJFA16, KJFA22, KJFA25, KJFA28, KJFA32, KJFA35, KJFA38, KJFA42, KJFA45, KJFA48, KJFA52, KJFA58, KJFA60, KJFA63, KJFA65, KJFA68, KJFA70, KJFA73, KJFA76, KJFA55, KJFA82, KJFA98,				

(*Continues*)

 © 2024. The Author(s).

Supplementary Table S2 (*Continued*)

	ICD 10	ICD 8	ATC codes	Definition	Registry
	KJGA00, KJGA02, KJGA05, KJGA22, KJGA28, KJGA32, KJGA35, KJGA52, KJGA58, KJGA60, KJGA70, KJGA73 KJGA75, KJGA76, KJGA96, KJGA97, KJGA98				
Open	Surgical codes: KJFH00, KJFH10, KJFH20, KJFH22, KJFH23, KJFH30, KJFH33, KJFH40, KJFH96, KJFB00, KJFB10, KJFB13, KJFB20, KJFB30, KJFB33, KJFB40, KJFB43, KJFB46, KJFB50, KJFB53, KJFB56, KJFB60, KJFB63, KJFB96, KJGB00, KJGB06, KJGB10, KJGB20, KJGB30, KJGB32, KJGB35, KJGB36, KJGB40, KJGB50, KJGB60, KJGB96, KJFA80, KJFA83, KJFA86, KJFA96.				
Percutaneous coronary intervention (PCI)/coronary artery bypass grafting (CABG)	Surgical codes: KFNA, KFNB, KFNC, KFND, KFNE, KFNF, KFNG, KFNH, KFNW, KFLF			Surgeries performed any time before CRC surgery	Danish National Patient Registry
Thrombolysis/thrombectomy for stroke	Surgical codes: KAAL10, KAAL11			Surgeries performed any time before CRC surgery	Danish National Patient Registry
Lower limb revascularization or amputation	Surgical codes:<br>KPDE, KPDF, KPDH, KPDN, KPDP, KPDQ KPEE, KPEF, KPEH, KPEN, KPEP, KPEQ, KPEU74, KPEU82, KPEU83, KPEU84 KPFE, KPFH, KPFN, KPFP, KPFQ, KPFU74, KPFU84.<br>KNFQ, KNGQ, KNHQ			Surgeries performed any time before CRC surgery	Danish National Patient Registry
Angina pectoris	I20, I251, I259	411, 413		Primary or secondary discharge diagnosis codes recorded during an inpatient hospitalization, emergency department, or outpatient clinic contact any time before CRC surgery	Danish National Patient Registry
Cardiovascular disease	Composite variable of myocardial infarction, cerebrovascular disease, congestive heart failure, and peripheral artery disease (see codes in the Charlson Comorbidity Index), as well as angina pectoris, PCI/CABG, thrombolysis/thrombectomy for stroke and lower limb revascularization or amputation			Primary or secondary discharge diagnosis codes recorded during an inpatient hospitalization, emergency department, or outpatient clinic contact any time before CRC surgery	Danish National Patient Registry

 © 2024. The Author(s).

Supplementary Table S2 (*Continued*)

	ICD 10	ICD 8	ATC codes	Definition	Registry
Hypertension	I10–I15	401, 402, 403, 404		1) ≥1 primary or secondary discharge diagnosis code<br>2) ≥2 different antihypertensive drug class prescriptions (angiotensin-converting enzyme inhibitors and angiotensin receptor blockers; β-blockers; Ca antagonists; non-loop diuretics; potassium-sparing agents; α-adrenergic antihypertensive agents)	Danish National Patient Registry
Smoking	Z587, Z720, J40–J44	491, 492	R03BB, R03AC, R03AK, R03CC, R03DB, R03DA, R03AL, R03BA	1) Primary or secondary discharge diagnosis codes recorded during an inpatient hospitalization, emergency department, or outpatient clinic contact any time before CRC surgery<br>2) ≥1 prescription for bronchodilators	Danish National Patient Registry and Danish National Prescription Registry
Arterial fibrillation	I48	427.92		Primary or secondary discharge diagnosis codes recorded during an inpatient hospitalization, emergency department, or outpatient clinic contact any time before CRC surgery	Danish National Patient Registry
Obesity	E65, E66, E68	27799		Primary or secondary discharge diagnosis codes recorded during an inpatient hospitalization, emergency department, or outpatient clinic contact any time before CRC surgery	Danish National Patient Registry
Chronic liver disease	See Charlson Comorbidity Index (encompasses both mild and severe liver disease)			Primary or secondary discharge diagnosis codes recorded during an inpatient hospitalization, emergency department, or outpatient clinic contact any time before CRC surgery	Danish National Patient Registry

(*Continues*)

 © 2024. The Author(s).

Supplementary Table S2 (*Continued*)

	ICD 10	ICD 8	ATC codes	Definition	Registry
Alcohol use disorder	F10, G31.2, G62.1, G72.1, I42.6, K29.2, K70, T51, R78.0, Z71.4 K86.0, Z72.1, E24.4, E52.9A, K85.2, L27.8A, Z50.2, Z71.4, BRHE2	291.00–291.99, 303.00–303.99, 577.10, 979, 980	N07BB	Primary or secondary discharge diagnosis codes recorded during an inpatient hospitalization, emergency department, or outpatient clinic contact any time before CRC surgery or a prescription for disulfiram	Danish National Patient Registry and Danish National Prescription Registry
Prior bleeding episodes	R04.0, R04.2, I85.0, K25.0, K25.2, K25.4, K25.6, K26.0, K26.2, K26.4, K26.6, K27.0, K27.2, K27.4, K27.6, K28.0, K28.2, K28.4, K28.6, K29.0, K62.5, K92.0–K92.2, K638C, R31.9, N02, D62, I60, I61, I62, S06.4–S06.6	430–431, 530.98, 783.09, 783.19, 531.90, 531.92, 531.95, 532.90, 533.90, 534.90, 535.01, 456.01, 569.15, 789.39		Primary or secondary discharge diagnosis codes recorded during an inpatient hospitalization, emergency department, or outpatient clinic contact any time before CRC surgery	Danish National Patient Registry
Prior venous thromboembolic events	I80.1–I80.3, I26	45100, 45099		Primary or secondary discharge diagnosis codes recorded during an inpatient hospitalization, emergency department, or outpatient clinic contact any time before CRC surgery	Danish National Patient Registry
Neoadjuvant chemotherapy	BOHJ, BWHA, BWHB, BWHC, BOHE, BJCZ01, BJHE11, BJHE12			Treatments received between diagnosis of CRC and the date of surgery	Danish National Patient Registry
Chronic kidney failure	E102, E112, DE122, E132, E142, I12, I13, I132, N083, N06, N00–N05, N07, N11, N14, N17, N18, N19, R809, Z992, Q61, BJFD	25002, 24902, 403, 404, 580–583, 584, 590.09, 593.19, 753.10–753.19, 792		Primary or secondary discharge diagnosis codes recorded during an inpatient hospitalization, emergency department, or outpatient clinic contact any time before CRC surgery	Danish National Patient Registry

Abbreviations: ATC, Anatomical Therapeutic Chemical; ICD, International Classification of Diseases.

 © 2024. The Author(s).

Supplementary Table S3 ATC codes for use of medication within 1 year before the date of colorectal cancer surgery ascertained from the Danish National Prescription Registry

Medication	ATC codes
Platelet aggregation inhibitors (aspirin and other platelet aggregation inhibitors)	B01AC06, N02BA01, N02BA51, B01AC04, B01AC22, B01AC07, B01AC24, B01AC25
Aspirin	B01AC06, N02BA01, N02BA51
Adenosine-diphosphate receptor antagonists	B01AC04, B01AC22, B01AC07, B01AC24, B01AC25
Thromboprophylaxis (vitamin K antagonists, DOACs, and heparins)	B01AA03, B01AA04, B01AE07, B01AF01, B01AF02, B01AF03, B01AX05, B01AB04, B01AB10, B01AB05, B01AB01
Vitamin K antagonists	B01AA03, B01AA04
DOACs (factor X inhibitors)	B01AE07, B01AF01, B01AF02, B01AF03, B01AX05
Heparins	B01AB04, B01AB10, B01AB05, B01AB01
Corticosteroids	H02AB, M01BA, A07EA
Non-aspirin NSAIDs	M01A
Antidepressants including SSRIs	N06A
Insulin	A10A
Oral glucose-lowering drugs	A10B
Statins	C10AA, C10BA, C10BX
Angiotensin-converting enzyme inhibitors and angiotensin receptor blockers	C09
Ca antagonists	C08
Beta-blockers	C07
Alpha-adrenergic antihypertensives	C02A, C02B, C02C
Loop diuretics	C03EB, C03C
Thiazides	C02DA, C02L, C03A, C03B,
Potassium-sparing agents	C03D C03EA, C03X
Proton pump inhibitors	A02BC01, A02BC02, A02BC03, A02BC04, A02BC05

Abbreviations: ATC, Anatomical Therapeutic Chemical; DOAC, direct oral anticoagulant; NSAID, nonsteroidal anti-inflammatory drug; SSRI, selective serotonin reuptake inhibitor.

Supplementary Table S4 Conditions included in the Charlson Comorbidity Index

Disease	ICD-8	ICD-10	Point
Myocardial infarction	410	I21, I22, I23	1
Congestive heart failure	427.09, 427.10, 427.11, 427.19, 428.99, 782.49	I50, I11.0, I13.0, I13.2	1
Peripheral vascular disease	440, 441, 442, 443, 444, 445	I70, I71, I72, I73, I74, I77	1
Cerebrovascular disease	430–438	I60–I69, G45, G46	1
Dementia	290.09–290.19, 293.09	F00–F03, F05.1, G30	1
Chronic pulmonary disease	490–493, 515–518	J40–J47, J60–J67, J68.4, J70.1, J70.3, J84.1, J92.0, J96.1, J98.2, J98.3	1
Connective tissue disease	712, 716, 734, 446, 135.99	M05, M06, M08, M09, M30, M31, M32, M33, M34, M35, M36, D86	1
Ulcer disease	530.91, 530.98, 531–534	K22.1, K25–K28	1

(*Continues*)

 © 2024. The Author(s).

Supplementary Table S4 (*Continued*)

Disease	ICD-8	ICD-10	Point
Mild liver disease	571, 573.01, 573.04	B18, K70.0–K70.3, K70.9, K71, K73, K74, K76.0	1
Diabetes type 1 and 2	Not included	Not included	Not included
Hemiplegia	344	G81, G82	2
Moderate-to-severe renal disease	403, 404, 580–583, 584, 590.09, 593.19, 753.10–753.19, 792	I12, I13, N00–N05, N07, N11, N14, N17–N19, Q61	2
Diabetes with end-organ damage	Not included	Not included	Not included
Any tumor (except CRC)	140–152, 155–194	C00–C75 (except C18–C20)	2
Leukemia	204–207	C91–C95	2
Lymphoma	200–203, 275.59	C81–C85, C88, C90, C96	2
Moderate to severe liver disease	070.00, 070.02, 070.04, 070.06, 070.08, 573.00, 456.00–456.09	B15.0, B16.0, B16.2, B19.0, K70.4, K72, K76.6, I85	3
Metastatic solid tumor	195–198, 199	C76–C80	6
AIDS	079.83	B21–B24	6

Abbreviations: AIDS, acquired immune deficiency syndrome; CRC, colorectal cancer; ICD, International Classification of Diseases.

Supplementary Table S5 Characteristics at the time of colorectal cancer surgery for patients with type 2 diabetes or without diabetes

	Type 2 diabetes	No diabetes
N (%)	6,305 (100.0%)	41,990 (100.0%)
Male (%)	3,910 (62.0%)	21,735 (52.0%)
Median age (Q1–Q3)	73.4 (67.3–79.4)	71.4 (63.7–78.7)
Age groups		
40–49	10 (0.0%)	405 (1.0%)
50–59	55 (1.0%)	1,455 (3.0%)
60–69	420 (7.0%)	5,105 (12.0%)
70–79	1,720 (27.0%)	11,805 (28.0%)
80+	2,640 (42.0%)	14,340 (34.0%)
Calendar year		
2005–2009	1,545 (24.0%)	12,695 (30.0%)
2010–2013	2,085 (33.0%)	13,705 (33.0%)
2014–2019	2,675 (42.0%)	15,585 (37.0%)
Median days from diagnosis of CRC until surgery (Q1–Q3)	6.0 (1.0–20.0)	6.0 (1.0–19.0)
Tumor stage		
Localized	2,765 (44.0%)	18,020 (43.0%)
Regional	1,585 (25.0%)	10,905 (26.0%)
Metastatic	915 (15.0%)	6,535 (16.0%)
Unknown	1,040 (17.0%)	6,530 (16.0%)
Type of first surgical approach		
Open	2,380 (38.0%)	15,730 (37.0%)
Laparoscopic	2,375 (38.0%)	15,875 (38.0%)
Endoscopic	1,555 (25.0%)	10,385 (25.0%)
Tumor resection site		

 © 2024. The Author(s).

Supplementary Table S5 (*Continued*)

	Type 2 diabetes	No diabetes
Total colectomy	155 (2.0%)	1,020 (2.0%)
Partial colectomy	3,475 (55.0%)	21,885 (52.0%)
Rectal resection	1,120 (18.0%)	8,665 (21.0%)
Other colorectal surgeries	1,555 (25.0%)	10,420 (25.0%)
Neoadjuvant chemotherapy	510 (8.0%)	3,215 (8.0%)
Hypertension	4,625 (73.0%)	16,165 (39.0%)
Obesity	1,140 (18.0%)	1,765 (4.0%)
Alcohol use disorder	450 (7.0%)	1,920 (5.0%)
Smoking	800 (13.0%)	3,595 (9.0%)
Comorbidities included in CCI		
Myocardial infarction	670 (11.0%)	2,185 (5.0%)
Congestive heart failure	690 (11.0%)	1,735 (4.0%)
Peripheral vascular disease	655 (10.0%)	2,085 (5.0%)
Cerebrovascular disease	1,015 (16.0%)	4,005 (10.0%)
Dementia	90 (1.0%)	420 (1.0%)
Chronic pulmonary disease	850 (13.0%)	3,965 (9.0%)
Connective tissue disease	280 (4.0%)	1,490 (4.0%)
Ulcer disease	445 (7.0%)	2,005 (5.0%)
Mild liver disease	160 (3.0%)	425 (1.0%)
Hemiplegia	15 (0.0%)	100 (0.0%)
Moderate-to-severe renal disease	365 (6.0%)	870 (2.0%)
Any tumor (except CRC)	940 (15.0%)	5,650 (13.0%)
Leukemia	25 (0.0%)	150 (0.0%)
Lymphoma	55 (1.0%)	395 (1.0%)
Moderate to severe liver disease	45 (1.0%)	125 (0.0%)
Metastatic solid tumor	140 (2.0%)	1,010 (2.0%)
AIDS	0 (0.0%)	20 (0.0%)
CCI score		
0	2,620 (42.0%)	24,375 (58.0%)
1–2	2,495 (40.0%)	13,055 (31.0%)
3+	1,190 (19.0%)	4,560 (11.0%)
Other comorbidities		
Prior bleeding episodes	1,460 (23.0%)	7,225 (17.0%)
Prior VTE	325 (5.0%)	1,770 (4.0%)
Angina pectoris	1,640 (26.0%)	5,100 (12.0%)
Percutaneous coronary revascularization	790 (13.0%)	2,170 (5.0%)
Lower limb revascularization or amputation	240 (4.0%)	525 (1.0%)
Atrial fibrillation	1,130 (18.0%)	4,160 (10.0%)
Chronic kidney failure	905 (14.0%)	1,735 (4.0%)
Medications		
Platelet aggregation inhibitors	2,990 (47.0%)	9,530 (23.0%)
Aspirin	2,730 (43.0%)	8,400 (20.0%)
Adenosine-diphosphate receptor antagonists	665 (11.0%)	2,585 (6.0%)

(*Continues*)

 © 2024. The Author(s).

Supplementary Table S5 (*Continued*)

	Type 2 diabetes	No diabetes
Thromboprophylaxis	930 (15.0%)	3,400 (8.0%)
Vitamin K antagonists	665 (11.0%)	2,415 (6.0%)
DOACs	300 (5.0%)	1,105 (3.0%)
Heparins	10 (0.0%)	75 (0.0%)
Non-aspirin NSAIDs	1,405 (22.0%)	8,765 (21.0%)
Antidepressants	895 (14.0%)	4,445 (11.0%)
Corticosteroids	500 (8.0%)	2,890 (7.0%)
Statins	4,200 (67.0%)	10,295 (25.0%)
Loop diuretics	1,550 (25.0%)	4,030 (10.0%)
ACE inhibitors/ARBs	4,165 (66.0%)	12,495 (30.0%)
Thiazides	1,330 (21.0%)	6,385 (15.0%)
Ca antagonists	2,205 (35.0%)	7,720 (18.0%)
Beta-blockers	2,200 (35.0%)	7,775 (19.0%)
Alpha-adrenergic antihypertensives	160 (3.0%)	375 (1.0%)
Potassium-sparing agents	535 (9.0%)	1,535 (4.0%)
Proton pump inhibitors	2,130 (34.0%)	10,465 (25.0%)

Abbreviations: ACE inhibitors/ARBs, angiotensin converting enzyme inhibitors/angiotensin receptor blockers; CCI, Charlson Comorbidity Index; CRC, colorectal cancer; DOACs, direct-acting oral anticoagulants; NSAIDs, nonsteroidal anti-inflammatory drugs; VTE, venous thromboembolism.
Note: Definitions of covariates are provided in ►**Supplementary Table S1–S4**. Numbers have been rounded to the nearest 5, in accordance with Danish health data legislation.

Supplementary Table S6 Risk and risk difference by subtypes of bleeding and venous thromboembolism for patients with type 2 diabetes or without diabetes undergoing colorectal cancer surgery

		N/events	Risk (95% CI)	Risk difference (95% CI)
Bleeding				
30 days	No diabetes	41,990/1,925	4.6 (4.4–4.8)	
	Type 2 diabetes	6,305/375	5.9 (5.4–6.5)	1.3 (0.2–2.6)
1 year	No diabetes	41,990/4,440	10.6 (10.3–10.9)	
	Type 2 diabetes	6,305/855	13.6 (12.8–14.4)	3.0 (1.3–4.8)
31–365 days	No diabetes	39,480/1,340	3.4 (3.2–3.6)	
	Type 2 diabetes	5,785/260	4.5 (4.0–5.0)	1.1 (0.0–2.2)
Respiratory tract bleeding				
30 days	No diabetes	41,990/20	0.0 (0.0–0.1)	
	Type 2 diabetes	6,305/0	0.0 (0.0–0.1)	−0.0 (−0.1–0.1)
1 year	No diabetes	41,990/130	0.3 (0.3–0.4)	
	Type 2 diabetes	6,305/35	0.6 (0.4–0.8)	0.2 (−0.1–0.7)
31–365 days	No diabetes	39,480/130	0.3 (0.3–0.4)	
	Type 2 diabetes	5,785/40	0.7 (0.5–1.0)	0.4 (−0.0–0.9)
Upper gastrointestinal bleeding				
30 days	No diabetes	41,990/80	0.2 (0.2–0.2)	
	Type 2 diabetes	6,305/20	0.3 (0.2–0.5)	0.1 (−0.1–0.5)
1 year	No diabetes	41,990/175	0.4 (0.4–0.5)	
	Type 2 diabetes	6,305/40	0.6 (0.5–0.9)	0.2 (−0.2–0.7)

 © 2024. The Author(s).

Supplementary Table S6 (*Continued*)

		N/events	Risk (95% CI)	Risk difference (95% CI)
31–365 days	No diabetes	39,480/105	0.3 (0.2–0.3)	
	Type 2 diabetes	5,785/20	0.4 (0.2–0.6)	0.1 (−0.2–0.5)
Lower gastrointestinal bleeding				
30 days	No diabetes	41,990/400	1.0 (0.9–1.1)	
	Type 2 diabetes	6,305/70	1.1 (0.9–1.4)	0.2 (−0.4–0.7)
1 year	No diabetes	41,990/930	2.3 (2.2–2.5)	
	Type 2 diabetes	6,305/175	3.0 (2.6–3.4)	0.7 (−0.2–1.6)
31–365 days	No diabetes	39,480/595	1.5 (1.4–1.6)	
	Type 2 diabetes	5,785/120	2.1 (1.8–2.5)	0.6 (−0.1–1.4)
Urinary tract bleeding				
30 days	No diabetes	41,990/45	0.1 (0.1–0.1)	
	Type 2 diabetes	6,305/10	0.2 (0.1–0.3)	0.1 (−0.1–0.3)
1 year	No diabetes	41,990/320	0.8 (0.7–0.9)	
	Type 2 diabetes	6,305/50	0.9 (0.7–1.1)	0.1 (−0.4–0.6)
31–365 days	No diabetes	39,480/295	0.8 (0.7–0.8)	
	Type 2 diabetes	5,785/40	0.7 (0.5–1.0)	−0.0 (−0.5–0.5)
Anemia from bleeding				
30 days	No diabetes	41,990/35	0.1 (0.1–0.1)	
	Type 2 diabetes	6,305/10	0.2 (0.1–0.3)	0.1 (−0.1–0.4)
1 year	No diabetes	41,990/85	0.2 (0.2–0.3)	
	Type 2 diabetes	6,305/20	0.3 (0.2–0.5)	0.1 (−0.1–0.5)
31–365 days	No diabetes	39,480/60	0.2 (0.1–0.2)	
	Type 2 diabetes	5,785/10	0.2 (0.1–0.4)	0.1 (−0.1–0.4)
Intracerebral bleeding				
30 days	No diabetes	41,990/15	0.0 (0.0–0.1)	
	Type 2 diabetes	6,305/0	0.0 (0.0–0.1)	−0.0 (−0.1–0.1)
1 year	No diabetes	41,990/95	0.2 (0.2–0.3)	
	Type 2 diabetes	6,305/20	0.4 (0.2–0.5)	0.1 (−0.2–0.5)
31–365 days	No diabetes	39,480/95	0.2 (0.2–0.3)	
	Type 2 diabetes	5,785/20	0.3 (0.2–0.5)	0.1 (−0.2–0.5)
Reoperation due to bleeding				
30 days	No diabetes	41,990/155	0.4 (0.3–0.4)	
	Type 2 diabetes	6,305/35	0.5 (0.4–0.8)	0.2 (−0.2–0.6)
1 year	No diabetes	41,990/195	0.5 (0.4–0.5)	
	Type 2 diabetes	6,305/40	0.6 (0.4–0.8)	0.1 (−0.2–0.6)
31–365 days	No diabetes	39,480/65	0.2 (0.1–0.2)	
	Type 2 diabetes	5,785/5	0.1 (0.0–0.2)	−0.1 (−0.2–0.2)
Venous thromboembolism				
30 days	No diabetes	41,990/285	0.7 (0.6–0.8)	
	Type 2 diabetes	6,305/40	0.7 (0.5–0.9)	−0.0 (−0.4–0.4)
1 year	No diabetes	41,990/1175	2.8 (2.6–3.0)	
	Type 2 diabetes	6,305/175	2.8 (2.4–3.2)	−0.0 (−0.8–0.9)
31–365 days	No diabetes	40,040/775	1.9 (1.8–2.1)	

(*Continues*)

 © 2024. The Author(s).

Supplementary Table S6 (*Continued*)

		N/events	Risk (95% CI)	Risk difference (95% CI)
	Type 2 diabetes	5,910/115	1.9 (1.6–2.3)	−0.0 (−0.7–0.8)
Deep venous thromboembolism				
30 days	No diabetes	41,990/70	0.2 (0.1–0.2)	
	Type 2 diabetes	6,305/5	0.1 (0.0–0.2)	−0.1 (−0.2–0.1)
1 year	No diabetes	41,990/360	0.9 (0.8–1.0)	
	Type 2 diabetes	6,305/45	0.7 (0.5–1.0)	−0.1 (−0.5–0.4)
31–365 days	No diabetes	40,040/295	0.7 (0.7–0.8)	
	Type 2 diabetes	5,910/45	0.7 (0.5–1.0)	−0.0 (−0.4–0.5)
Pulmonary embolism				
30 days	No diabetes	41,990/170	0.4 (0.3–0.5)	
	Type 2 diabetes	6,305/30	0.5 (0.4–0.7)	0.1 (−0.2–0.5)
1 year	No diabetes	41,990/645	1.5 (1.4–1.7)	
	Type 2 diabetes	6,305/100	1.6 (1.3–2.0)	0.1 (−0.5–0.8)
31–365 days	No diabetes	40,040/480	1.2 (1.1–1.3)	
	Type 2 diabetes	5,910/70	1.2 (1.0–1.5)	−0.0 (−0.5–0.6)

Abbreviations: CI, confidence interval.
Note: The overall results are depicted in ►**Table 2**. Patients were followed from their surgery date until an event of interest, emigration, death, or study end (April 1, 2021), whichever came first. The Aalen–Johansen estimator was used to estimate 30-day and 1-year risk of bleeding and venous thromboembolism, by considering the competing risk of death. Hazard ratios were not estimated for each sub-outcome because of too few events.

Supplementary Table S7 1-year absolute risk, risk difference, and adjusted SHR for bleeding in patients with type 2 diabetes or without diabetes undergoing colorectal cancer surgery, stratified by patient characteristics

Subgroup		*N*/events	Risk (95% CI)	Risk difference (95% CI)	Age-, sex-, and calendar- year-adjusted SHR (95% CI)
Overall	No diabetes	41,990/2,175	5.2 (5.0–5.4)		
	Type 2 diabetes	6,305/435	6.9 (6.3–7.5)	1.7 (0.4–3.0)	1.26 (1.14–1.40)
Age group[a]					
50–59	No diabetes	5,105/200	4.0 (3.4–4.5)		1.74 (1.18–2.58)
	Type 2 diabetes	420/30	6.9 (4.8–9.6)	3.0 (−1.4–8.4)	
60–69	No diabetes	11,805/510	4.3 (4.0–4.7)		1.36 (1.10–1.68)
	Type 2 diabetes	1,720/105	6.2 (5.1–7.4)	1.8 (−0.4–4.3)	
70–79	No diabetes	14,340/820	5.7 (5.4–6.1)		1.08 (0.91–1.28)
	Type 2 diabetes	2,640/165	6.3 (5.4–7.3)	0.6 (−1.3–2.6)	
80+	No diabetes	8,875/560	6.3 (5.8–6.8)		1.35 (1.11–1.64)
	Type 2 diabetes	1,465/125	8.6 (7.2–10.1)	2.3 (−0.6–5.4)	
Sex					
Female	No diabetes	20,250/850	4.2 (3.9–4.5)		1.40 (1.17–1.67)
	Type 2 diabetes	2,395/145	6.0 (5.1–7.0)	1.8 (−0.1–3.8)	
Male	No diabetes	21,735/1,325	6.1 (5.8–6.4)		1.20 (1.06–1.37)
	Type 2 diabetes	3,910/290	7.4 (6.6–8.3)	1.3 (−0.3–3.1)	
Calendar year					
2005–2009	No diabetes	12,695/675	5.3 (4.9–5.7)		1.23 (1.00–1.50)
	Type 2 diabetes	1,545/110	7.1 (5.9–8.4)	1.7 (−0.8–4.5)	

 © 2024. The Author(s).

Supplementary Table S7 (*Continued*)

Subgroup		*N*/events	Risk (95% CI)	Risk difference (95% CI)	Age-, sex-, and calendar- year-adjusted SHR (95% CI)
2010–2014	No diabetes	13,705/725	5.3 (4.9–5.7)		1.18 (0.98–1.42)
	Type 2 diabetes	2,085/140	6.6 (5.6–7.7)	1.3 (−0.8–3.6)	
2015–2019	No diabetes	15,585/775	5.0 (4.6–5.3)		1.37 (1.16–1.61)
	Type 2 diabetes	2,675/185	7.0 (6.1–8.0)	2.0 (0.1–4.1)	
Tumor stage					
Localized	No diabetes	18,020/910	5.0 (4.7–5.4)		1.32 (1.13–1.55)
	Type 2 diabetes	2,765/195	7.0 (6.1–8.0)	2.0 (0.1–4.0)	
Regional	No diabetes	10,905/510	4.7 (4.3–5.1)		1.36 (1.10–1.68)
	Type 2 diabetes	1,585/105	6.6 (5.5–7.9)	2.0 (−0.4–4.6)	
Metastatic	No diabetes	6,535/380	5.8 (5.3–6.4)		1.20 (0.92–1.56)
	Type 2 diabetes	915/65	7.3 (5.8–9.1)	1.5 (−1.8–5.2)	
Unknown	No diabetes	6,530/380	5.8 (5.3–6.4)		1.07 (0.82–1.39)
	Type 2 diabetes	1,040/65	6.4 (5.1–8.0)	0.6 (−2.3–3.9)	
Type of first surgical approach					
Open	No diabetes	15,730/760	4.8 (4.5–5.2)		1.19 (1.00–1.43)
	Type 2 diabetes	2,380/145	6.1 (5.2–7.2)	1.3 (−0.6–3.4)	
Laparoscopic	No diabetes	15,875/770	4.8 (4.5–5.2)		1.34 (1.13–1.60)
	Type 2 diabetes	2,375/160	6.7 (5.7–7.7)	1.8 (−0.2–4.0)	
Endoscopic	No diabetes	10,385/645	6.2 (5.8–6.7)		1.28 (1.05–1.55)
	Type 2 diabetes	1,555/130	8.4 (7.1–9.8)	2.2 (−0.6–5.1)	
CCI score					
0	No diabetes	24,375/995	4.1 (3.8–4.3)		1.31 (1.10–1.56)
	Type 2 diabetes	2,620/145	5.5 (4.7–6.5)	1.4 (−0.3–3.3)	
1–2	No diabetes	13,055/815	6.2 (5.8–6.7)		1.07 (0.91–1.26)
	Type 2 diabetes	2,495/175	6.9 (6.0–8.0)	0.7 (−1.3–2.9)	
+3	No diabetes	4,560/365	8.0 (7.2–8.8)		1.21 (0.98–1.50)
	Type 2 diabetes	1,190/115	9.8 (8.2–11.5)	1.8 (−1.7–5.5)	
Chronic liver disease					
No	No diabetes	41,505/2,140	5.2 (5.0–5.4)		1.24 (1.11–1.38)
	Type 2 diabetes	6,135/415	6.7 (6.1–7.4)	1.6 (0.3–2.9)	
Yes	No diabetes	485/35	6.8 (4.8–9.3)		1.73 (0.98–3.04)
	Type 2 diabetes	175/20	12.1 (7.8–17.5)	5.3 (−4.5–16.5)	
Chronic kidney disease					
No	No diabetes	40,805/2,070	5.1 (4.9–5.3)		1.24 (1.11–1.38)
	Type 2 diabetes	5,660/375	6.6 (6.0–7.3)	1.5 (0.2–2.9)	
Yes	No diabetes	1,180/105	8.9 (7.4–10.6)		1.10 (0.79–1.52)
	Type 2 diabetes	650/60	9.4 (7.3–11.8)	0.5 (−4.8–6.1)	
Hypertension					
No	No diabetes	25,820/1,110	4.3 (4.0–4.5)		1.36 (1.11–1.67)
	Type 2 diabetes	1,680/105	6.1 (5.0–7.3)	1.8 (−0.3–4.3)	
Yes	No diabetes	16,165/1,065	6.6 (6.2–7.0)		1.06 (0.94–1.20)
	Type 2 diabetes	4,625/330	7.2 (6.4–7.9)	0.6 (−1.0–2.2)	

(*Continues*)

 © 2024. The Author(s).

Supplementary Table S7 (*Continued*)

Subgroup		*N*/events	Risk (95% CI)	Risk difference (95% CI)	Age-, sex-, and calendar- year-adjusted SHR (95% CI)
Obesity					
No	No diabetes	40,225/2,065	5.1 (4.9–5.4)		1.24 (1.11–1.39)
	Type 2 diabetes	5,170/350	6.8 (6.1–7.5)	1.7 (0.3–3.1)	
Yes	No diabetes	1,765/110	6.3 (5.2–7.5)		1.09 (0.81–1.47)
	Type 2 diabetes	1,140/85	7.3 (5.9–8.9)	1.0 (−2.6–4.8)	
Arterial fibrillation					
No	No diabetes	37,830/1,790	4.7 (4.5–4.9)		1.28 (1.13–1.44)
	Type 2 diabetes	5,175/325	6.3 (5.7–7.0)	1.6 (0.3–3.0)	
Yes	No diabetes	4,160/385	9.3 (8.4–10.2)		1.02 (0.82–1.27)
	Type 2 diabetes	1,130/105	9.5 (7.8–11.3)	0.2 (−3.5–4.1)	
Cardiovascular disease					
No	No diabetes	31,580/1,340	4.2 (4.0–4.5)		1.35 (1.17–1.57)
	Type 2 diabetes	3,475/205	5.9 (5.1–6.7)	1.7 (0.1–3.3)	
Yes	No diabetes	10,405/835	8.0 (7.5–8.5)		0.99 (0.85–1.15)
	Type 2 diabetes	2,830/230	8.1 (7.1–9.1)	0.1 (−2.1–2.3)	
Prior bleeding					
No	No diabetes	34,765/1,450	4.2 (4.0–4.4)		1.21 (1.06–1.38)
	Type 2 diabetes	4,845/255	5.3 (4.7–6.0)	1.1 (−0.1–2.5)	
Yes	No diabetes	7,225/725	10.0 (9.3–10.7)		1.20 (1.02–1.42)
	Type 2 diabetes	1,460/175	12.1 (10.5–13.8)	2.1 (−1.4–5.7)	
Prior VTEs					
No	No diabetes	40,220/2,065	5.1 (4.9–5.4)		1.29 (1.16–1.43)
	Type 2 diabetes	5,985/415	7.0 (6.3–7.6)	1.8 (0.5–3.2)	
Yes	No diabetes	1,770/110	6.2 (5.1–7.3)		0.83 (0.51–1.37)
	Type 2 diabetes	325/20	5.6 (3.4–8.4)	-0.6 (-5.4–5.4)	
Platelet aggregation inhibitors					
No	No diabetes	32,455/1,410	4.3 (4.1–4.6)		1.42 (1.23–1.65)
	Type 2 diabetes	3,315/210	6.4 (5.6–7.3)	2.1 (0.4–3.8)	
Yes	No diabetes	9,530/765	8.0 (7.5–8.6)		0.90 (0.77–1.05)
	Type 2 diabetes	2,990/220	7.4 (6.5–8.4)	-0.6 (-2.7–1.6)	
Thromboprophylaxis					
No	No diabetes	38,585/1,845	4.8 (4.6–5.0)		1.25 (1.11–1.41)
	Type 2 diabetes	5,380/335	6.2 (5.6–6.9)	1.4 (0.1–2.8)	
Yes	No diabetes	3,400/330	9.7 (8.7–10.7)		1.09 (0.87–1.37)
	Type 2 diabetes	930/100	10.7 (8.8–12.7)	1.0 (-3.2–5.5)	
Statin					
No	No diabetes	31,690/1,470	4.6 (4.4–4.9)		1.47 (1.24–1.74)
	Type 2 diabetes	2,110/155	7.3 (6.2–8.4)	2.6 (0.5–4.9)	
Yes	No diabetes	1,0295/705	6.8 (6.4–7.3)		0.96 (0.84–1.11)
	Type 2 diabetes	4,200/280	6.7 (6.0–7.5)	-0.1 (-1.9–1.7)	

Abbreviations: CCI, Charlson Comorbidity index; CI, confidence interval; SHR, subdistribution hazard ratio; VTE, venous thromboembolism.
Note: Patients were followed from their surgery date until the first occurrence of a hospital-diagnosed bleeding event, emigration, death, or study end (April 1, 2021), whichever came first. The Aalen–Johansen estimator was used to estimate 30-day and 1-year risks of bleeding, by considering competing risk of death.
[a]The age group <50 years was too small and had too few events to constitute a meaningful subgroup.

 © 2024. The Author(s).

Supplementary Table S8 1-year absolute risk, risk difference, and adjusted SHR for venous thromboembolic events in patients with type 2 diabetes or without diabetes undergoing CRC surgery, stratified by patient characteristics

Subgroup		*N*/events	Risk (95% CI)	Risk difference (95% CI)	Age-, sex-, and calendar year adjusted SHR (95% CI)
Overall	No diabetes	41,990/1,020	2.4 (2.3–2.6)		
	Type 2 diabetes	6,305/150	2.4 (2.0–2.8)	−0.0 (−0.8–0.8)	0.96 (0.81–1.14)
Age group[a]					
50–59	No diabetes	5,105/100	2.0 (1.6–2.4)		1.39 (0.76–2.53)
	Type 2 diabetes	420/10	2.9 (1.6–4.8)	0.9 (−1.8–4.7)	
60–69	No diabetes	11,805/310	2.6 (2.3–2.9)		1.19 (0.89–1.60)
	Type 2 diabetes	1,720/55	3.2 (2.4–4.1)	0.6 (−1.0–2.4)	
70–79	No diabetes	14,340/395	2.8 (2.5–3.0)		0.81 (0.61–1.07)
	Type 2 diabetes	2,640/60	2.2 (1.7–2.9)	−0.5 (−1.7–0.8)	
80+	No diabetes	8,875/170	1.9 (1.7–2.2)		0.73 (0.47–1.16)
	Type 2 diabetes	1,465/20	1.4 (0.9–2.1)	−0.5 (−1.7–1.0)	
Sex					
Female	No diabetes	20,250/470	2.3 (2.1–2.5)		1.24 (0.96–1.59)
	Type 2 diabetes	2,395/70	3.0 (2.3–3.7)	0.6 (−0.7–2.1)	
Male	No diabetes	21,735/550	2.5 (2.3–2.7)		0.79 (0.63–1.01)
	Type 2 diabetes	3,910/80	2.0 (1.6–2.5)	−0.5 (−1.4–0.5)	
Calendar year					
2005–2009	No diabetes	12,695/255	2.0 (1.8–2.3)		0.81 (0.54–1.22)
	Type 2 diabetes	1,545/25	1.7 (1.1–2.4)	−0.3 (−1.5–1.2)	
2010–2014	No diabetes	13,705/355	2.6 (2.3–2.9)		1.00 (0.75–1.34)
	Type 2 diabetes	2,085/55	2.6 (2.0–3.4)	0.0 (−1.3–1.6)	
2015–2019	No diabetes	15,585/410	2.6 (2.4–2.9)		1.00 (0.77–1.29)
	Type 2 diabetes	2,675/70	2.6 (2.1–3.3)	−0.0 (−1.2–1.4)	
Tumor stage					
Localized	No diabetes	18,020/295	1.6 (1.5–1.8)		0.94 (0.68–1.29)
	Type 2 diabetes	2,765/45	1.6 (1.2–2.2)	−0.0 (−0.9–1.1)	
Regional	No diabetes	10,905/310	2.8 (2.5–3.2)		1.17 (0.87–1.58)
	Type 2 diabetes	1,585/55	3.4 (2.6–4.4)	0.6 (−1.1–2.6)	
Metastatic	No diabetes	6,535/255	3.9 (3.4–4.4)		0.98 (0.69–1.40)
	Type 2 diabetes	915/35	3.9 (2.8–5.3)	0.1 (−2.3–2.9)	
Unknown	No diabetes	6,530/165	2.5 (2.2–2.9)		0.61 (0.37–1.03)
	Type 2 diabetes	1,040/15	1.5 (0.9–2.4)	−1.0 (−2.4–0.9)	
Type of first surgical approach					
Open	No diabetes	15,730/425	2.7 (2.5–3.0)		0.85 (0.64–1.12)
	Type 2 diabetes	2,380/55	2.4 (1.8–3.0)	−0.4 (−1.6–1.0)	
Laparoscopic	No diabetes	15,875/320	2.0 (1.8–2.2)		1.17 (0.88–1.56)
	Type 2 diabetes	2,375/60	2.5 (1.9–3.2)	0.5 (−0.7–1.9)	
Endoscopic	No diabetes	10,385/275	2.7 (2.4–3.0)		0.86 (0.60–1.22)
	Type 2 diabetes	1,555/35	2.3 (1.7–3.2)	−0.3 (−1.8–1.4)	

(*Continues*)

 © 2024. The Author(s).

Supplementary Table S8 (*Continued*)

Subgroup		*N*/events	Risk (95% CI)	Risk difference (95% CI)	Age-, sex-, and calendar year adjusted SHR (95% CI)
CCI score					
0	No diabetes	24,375/595	2.4 (2.2–2.6)		1.16 (0.91–1.47)
	Type 2 diabetes	2,620/75	2.9 (2.3–3.6)	0.5 (−0.7–1.9)	
1–2	No diabetes	13,055/315	2.4 (2.2–2.7)		0.90 (0.67–1.20)
	Type 2 diabetes	2,495/55	2.2 (1.7–2.8)	−0.2 (−1.4–1.1)	
+3	No diabetes	4,560/110	2.5 (2.0–2.9)		0.64 (0.39–1.04)
	Type 2 diabetes	1,190/20	1.6 (1.0–2.4)	−0.9 (−2.4–1.0)	
Chronic liver disease					
No	No diabetes	41,505/1,010	2.4 (2.3–2.6)		0.96 (0.80–1.14)
	Type 2 diabetes	6,135/145	2.4 (2.0–2.8)	−0.0 (−0.8–0.8)	
Yes	No diabetes	485/15	2.7 (1.5–4.4)		1.09 (0.39–3.03)
	Type 2 diabetes	175/5	2.9 (1.1–6.2)	0.2 (−4.7–7.1)	
Chronic kidney disease					
No	No diabetes	40,805/990	2.4 (2.3–2.6)		0.96 (0.80–1.15)
	Type 2 diabetes	5,660/135	2.4 (2.0–2.8)	−0.0 (−0.8–0.8)	
Yes	No diabetes	1,180/30	2.6 (1.8–3.7)		0.89 (0.49–1.62)
	Type 2 diabetes	650/15	2.5 (1.5–3.9)	−0.2 (−3.0–3.0)	
Hypertension					
No	No diabetes	25,820/640	2.5 (2.3–2.7)		1.18 (0.88–1.57)
	Type 2 diabetes	1,680/50	3.0 (2.2–3.9)	0.5 (−1.0–2.3)	
Yes	No diabetes	16,165/385	2.4 (2.1–2.6)		0.90 (0.72–1.12)
	Type 2 diabetes	4,625/100	2.2 (1.8–2.6)	−0.2 (−1.1–0.8)	
Obesity					
No	No diabetes	40,225/970	2.4 (2.3–2.6)		0.91 (0.75–1.11)
	Type 2 diabetes	5,170/115	2.3 (1.9–2.7)	−0.1 (−0.9–0.8)	
Yes	No diabetes	1,765/50	2.9 (2.2–3.8)		1.08 (0.69–1.69)
	Type 2 diabetes	1,140/35	3.0 (2.1–4.1)	0.0 (−2.4–2.6)	
Arterial fibrillation					
No	No diabetes	37,830/935	2.5 (2.3–2.6)		1.04 (0.87–1.25)
	Type 2 diabetes	5,175/140	2.7 (2.3–3.1)	0.2 (−0.7–1.2)	
Yes	No diabetes	4,160/85	2.1 (1.7–2.5)		0.54 (0.30–0.97)
	Type 2 diabetes	1,130/15	1.1 (0.6–1.9)	−0.9 (−2.3–0.8)	
Cardiovascular disease					
No	No diabetes	31,580/805	2.5 (2.4–2.7)		1.09 (0.88–1.34)
	Type 2 diabetes	3,475/100	2.9 (2.4–3.5)	0.3 (−0.7–1.5)	
Yes	No diabetes	10,405/220	2.1 (1.8–2.4)		0.84 (0.62–1.15)
	Type 2 diabetes	2,830/50	1.8 (1.4–2.3)	−0.3 (−1.3–0.9)	
Prior bleeding					
No	No diabetes	34,765/870	2.5 (2.3–2.7)		0.98 (0.81–1.19)
	Type 2 diabetes	4,845/125	2.5 (2.1–3.0)	0.0 (−0.8–1.0)	
Yes	No diabetes	7,225/150	2.1 (1.8–2.4)		0.90 (0.60–1.34)
	Type 2 diabetes	1,460/30	1.9 (1.3–2.7)	−0.2 (−1.6–1.5)	

 © 2024. The Author(s).

Supplementary Table S8 (*Continued*)

Subgroup		*N*/events	Risk (95% CI)	Risk difference (95% CI)	Age-, sex-, and calendar year adjusted SHR (95% CI)
Prior VTEs					
No	No diabetes	40,220/755	1.9 (1.8–2.0)		0.98 (0.80–1.19)
	Type 2 diabetes	5,985/110	1.9 (1.6–2.2)	−0.0 (−0.7–0.8)	
Yes	No diabetes	1,770/265	14.9 (13.3–16.6)		0.80 (0.57–1.12)
	Type 2 diabetes	325/40	12.0 (8.8–15.8)	−2.9 (−10.1–5.2)	
Platelet aggregation inhibitors					
No	No diabetes	32,455/835	2.6 (2.4–2.8)		1.01 (0.81–1.26)
	Type 2 diabetes	3,315/90	2.7 (2.2–3.3)	0.1 (−0.9–1.3)	
Yes	No diabetes	9,530/185	1.9 (1.7–2.2)		1.03 (0.77–1.38)
	Type 2 diabetes	2,990/60	2.1 (1.6–2.6)	0.1 (−0.9–1.3)	
Thromboprophylaxis					
No	No diabetes	38,585/890	2.3 (2.2–2.5)		1.03 (0.86–1.24)
	Type 2 diabetes	5,380/135	2.5 (2.1–2.9)	0.2 (−0.7–1.1)	
Yes	No diabetes	3,400/130	3.8 (3.2–4.5)		0.53 (0.32–0.87)
	Type 2 diabetes	930/20	1.9 (1.2–3.0)	−1.9 (−3.8–0.5)	
Statin					
No	No diabetes	31,690/810	2.6 (2.4–2.7)		0.92 (0.69–1.22)
	Type 2 diabetes	2,110/50	2.4 (1.8–3.1)	−0.2 (−1.4–1.3)	
Yes	No diabetes	10,295/210	2.0 (1.8–2.3)		1.17 (0.92–1.49)
	Type 2 diabetes	4,200/100	2.4 (2.0–2.9)	0.4 (−0.6–1.5)	

Abbreviations: CCI, Charlson Comorbidity index; CI, confidence interval; VTE, venous thromboembolism; SHR, subdistribution hazard ratio.
Note: Patients were followed from their surgery date until the first occurrence of venous thromboembolism, emigration, death, or study end (April 1, 2021), whichever came first. The Aalen–Johansen estimator was used to estimate 30-day and 1-year risk of venous thromboembolism, by considering the competing risk of death.
[a]The age group <50 years was too small with too few events to form a meaningful subgroup.

Supplementary Table S9 1-year mortality risk among patients with a bleeding or VTE event 1 year after colorectal cancer surgery

Cohort		N/events	Risk (95% CI)	Adjusted cause-specific HR (95% CI)
Bleeding cohort	No diabetes	2,175/540	24.9 (23.2–26.8)	Reference
	Type 2 diabetes	435/115	26.0 (22.2–30.4)	1.46 (0.59–3.60)
VTE cohort	No diabetes	150/40	27.5 (24.9–30.4)	Reference
	Type 2 diabetes	1,020/280	25.8 (19.6–33.6)	1.02 (0.73–1.43)

Abbreviations: CI, confidence interval; HR, hazard ratio; VTE, venous thromboembolism.
Note: Patients were followed from bleeding/VTE episode until 1 year of follow-up, death, or study end (April 1, 2021), whichever came first. The Kaplan–Meier estimator was used to estimate 1-year mortality. HRs were adjusted for age, sex, and calendar year. Numbers have been rounded to the nearest 5, in accordance with Danish health data legislation.

 © 2024. The Author(s).

Supplementary Table S10 Risks, cause-specific HRs, and adjusted subdistribution HRs of bleeding and venous thromboembolism for patients with T2D or without diabetes undergoing CRC surgery

		N/events	Risk (95% CI)	Risk difference (95% CI)	Age-, sex-, and calendar year adjusted HR (95% CI)	Age-, sex-, and calendar year adjusted SHR (95% CI)
Bleeding events						
31–365 days	No diabetes	39,480/1,340	3.4 (3.2–3.6)		Reference	Reference
	Type 2 diabetes	5,785/260	4.5 (4.0–5.0)	1.1 (0.0–2.2)	1.34 (1.18–1.53)	1.24 (1.09–1.42)
Venous thromboembolism						
31–365 days	No diabetes	40,040/775	1.9 (1.8–2.1)		Reference	Reference
	Type 2 diabetes	5,910/115	1.9 (1.6–2.3)	−0.0 (−0.7–0.8)	0.97 (0.80–1.19)	0.97 (0.79–1.18)

Abbreviations: CRC, colorectal cancer; HR, hazard ratio; SHR, subdistribution hazard ratio.
Note: Patients were followed from the date of surgery until the first occurrence of an event of interest, emigration, death, or study end (April 1, 2021), whichever came first. The Aalen–Johansen estimator was used to estimate 30-day and 1-year risks of bleeding and venous thromboembolism, by considering the competing risk of death. The Fine and Gray regression model was used to estimate SHRs to account for competing risk. Numbers have been rounded to the nearest 5, in accordance with Danish health data legislation.

Supplementary Table S11 Risk, risk differences, and adjusted SHR for bleeding events and venous thromboembolic events in patients with type 2 diabetes or without diabetes undergoing colorectal cancer surgery, on the basis of more extensive outcome definitions

		N/events	Risk (95% CI)	Risk difference (95% CI)	Adjusted SHR (95% CI)
Bleeding					
30 days	No diabetes	41,990/1925	4.6 (4.4–4.8)		1.30 (1.16–1.45)
	Type 2 diabetes	6,305/375	5.9 (5.4–6.5)	1.3 (0.2–2.6)	
1 year	No diabetes	41,990/4440	10.6 (10.3–10.9)		1.31 (1.21–1.41)
	Type 2 diabetes	6,305/855	13.6 (12.8–14.4)	3.0 (1.3–4.8)	
Respiratory tract bleeding					
30 days	No diabetes	41,990/20	0.0 (0.0–0.1)		
	Type 2 diabetes	6,305/0	0.0 (0.0–0.1)	−0.0 (−0.1–0.1)	
1 year	No diabetes	41,990/130	0.3 (0.3–0.4)		
	Type 2 diabetes	6,305/35	0.6 (0.4–0.8)	0.2 (−0.1–0.7)	
Upper gastrointestinal bleeding					
30 days	No diabetes	41,990/80	0.2 (0.2–0.2)		
	Type 2 diabetes	6,305/20	0.3 (0.2–0.5)	0.1 (−0.1–0.5)	
1 year	No diabetes	41,990/175	0.4 (0.4–0.5)		
	Type 2 diabetes	6,305/40	0.6 (0.5–0.9)	0.2 (−0.2–0.7)	
Lower gastrointestinal bleeding					
30 days	No diabetes	41,990/400	1.0 (0.9–1.1)		
	Type 2 diabetes	6,305/70	1.1 (0.9–1.4)	0.2 (−0.4–0.7)	
1 year	No diabetes	41,990/930	2.3 (2.2–2.5)		
	Type 2 diabetes	6,305/175	3.0 (2.6–3.4)	0.7 (−0.2–1.6)	
Urinary tract bleeding					
30 days	No diabetes	41,990/45	0.1 (0.1–0.1)		
	Type 2 diabetes	6,305/10	0.2 (0.1–0.3)	0.1 (−0.1–0.3)	

 © 2024. The Author(s).

Supplementary Table S11 (*Continued*)

		N/events	Risk (95% CI)	Risk difference (95% CI)	Adjusted SHR (95% CI)
1 year	No diabetes	41,990/320	0.8 (0.7–0.9)		
	Type 2 diabetes	6,305/50	0.9 (0.7–1.1)	0.1 (−0.4–0.6)	
Anemia from bleeding					
30 days	No diabetes	41,990/35	0.1 (0.1–0.1)		
	Type 2 diabetes	6,305/10	0.2 (0.1–0.3)	0.1 (−0.1–0.4)	
1 year	No diabetes	41,990/85	0.2 (0.2–0.3)		
	Type 2 diabetes	6,305/20	0.3 (0.2–0.5)	0.1 (−0.1–0.5)	
Intracerebral bleeding					
30 days	No diabetes	41,990/15	0.0 (0.0–0.1)		
	Type 2 diabetes	6,305/0	0.0 (0.0–0.1)	−0.0 (−0.1–0.1)	
1 year	No diabetes	41,990/95	0.2 (0.2–0.3)		
	Type 2 diabetes	6,305/20	0.4 (0.2–0.5)	0.1 (−0.2–0.5)	
Reoperation due to bleeding					
30 days	No diabetes	41,990/155	0.4 (0.3–0.4)		
	Type 2 diabetes	6,305/35	0.5 (0.4–0.8)	0.2 (−0.2–0.6)	
1 year	No diabetes	41,990/195	0.5 (0.4–0.5)		
	Type 2 diabetes	6,305/40	0.6 (0.4–0.8)	0.1 (−0.2–0.6)	
Postoperative bleeding/hematoma					
30 days	No diabetes	41,990/215	0.5 (0.5–0.6)		
	Type 2 diabetes	6,305/40	0.7 (0.5–0.9)	0.1 (−0.2–0.6)	
1 year	No diabetes	41,990/365	0.9 (0.8–1.0)		
	Type 2 diabetes	6,305/65	1.1 (0.8–1.4)	0.2 (−0.3–0.8)	
Treatment with blood products					
30 days	No diabetes	41,990/960	2.3 (2.2–2.5)		
	Type 2 diabetes	6,305/185	3.0 (2.6–3.4)	0.7 (−0.2–1.6)	
1 year	No diabetes	41,990/2150	5.3 (5.0–5.5)		
	Type 2 diabetes	6,305/415	6.8 (6.2–7.5)	1.6 (0.3–2.9)	
Venous thromboembolism					
30 days	No diabetes	41,990/285	0.7 (0.6–0.8)		0.95 (0.69–1.32)
	Type 2 diabetes	6,305/40	0.7 (0.5–0.9)	−0.0 (−0.4–0.4)	
1 year	No diabetes	41,990/1175	2.8 (2.6–3.0)		0.98 (0.83–1.15)
	Type 2 diabetes	6,305/175	2.8 (2.4–3.2)	−0.0 (−0.8–0.9)	
Deep venous thromboembolism					
30 days	No diabetes	41,990/70	0.2 (0.1–0.2)		
	Type 2 diabetes	6,305/5	0.1 (0.0–0.2)	−0.1 (−0.2–0.1)	
1 year	No diabetes	41,990/360	0.9 (0.8–1.0)		
	Type 2 diabetes	6,305/45	0.7 (0.5–1.0)	−0.1 (−0.5–0.4)	
Pulmonary embolism					
30 days	No diabetes	41,990/170	0.4 (0.3–0.5)		
	Type 2 diabetes	6,305/30	0.5 (0.4–0.7)	0.1 (−0.2–0.5)	
1 year	No diabetes	41,990/645	1.5 (1.4–1.7)		
	Type 2 diabetes	6,305/100	1.6 (1.3–2.0)	0.1 (−0.5–0.8)	

(*Continues*)

 © 2024. The Author(s).

Supplementary Table S11 (*Continued*)

		N/events	Risk (95% CI)	Risk difference (95% CI)	Adjusted SHR (95% CI)
Postoperative thromboembolism					
30 days	No diabetes	41,990/20	0.0 (0.0–0.1)		
	Type 2 diabetes	6,305/5	0.1 (0.0–0.2)	0.0 (−0.1–0.2)	
1 year	No diabetes	41,990/50	0.1 (0.1–0.2)		
	Type 2 diabetes	6,305/10	0.1 (0.1–0.3)	0.0 (−0.1–0.3)	
Unspecified thrombosis					
30 days	No diabetes	41,990/25	0.1 (0.0–0.1)		
	Type 2 diabetes	6,305/0	0.0 (0.0–0.1)	−0.0 (−0.1–0.1)	
1 year	No diabetes	41,990/125	0.3 (0.3–0.4)		
	Type 2 diabetes	6,305/20	0.3 (0.2–0.5)	−0.0 (−0.3–0.3)	

Abbreviations: CI, confidence interval; HR, hazard ratio.
Note: Patients were followed from their surgery date until the first occurrence of an event of interest, emigration, death, or study end (April 1, 2021), whichever came first. The Aalen – Johansen estimator was used to estimate 30-day and 1-year risk of venous thromboembolism, by considering the competing risk of death. Adjusted HRs are shown for only the composite outcome, because of too few events for each sub-outcome.

 © 2024. The Author(s).
